# 6-Amino-2-(pivaloyl­amino)­pyridinium benzoate

**DOI:** 10.1107/S1600536813023787

**Published:** 2013-08-31

**Authors:** Lilianna Chęcińska, Borys Ośmiałowski, Arto Valkonen

**Affiliations:** a Structural Chemistry and Crystallography Group, University of Lodz, Pomorska 163/165, PL-90-236 Łódź, Poland; b Faculty of Technology and Chemical Engineering, University of Technology and Life Sciences, Seminaryjna 3, PL-85-326 Bydgoszcz, Poland; c Department of Chemistry, University of Jyväskylä, PO Box 35, FI-40014 Jyväskylä, Finland

## Abstract

In the crystal structure of the title salt, C_10_H_16_N_3_O^+^·C_7_H_5_O_2_^−^, the cations and anions are linked to each other *via* N—H⋯O hydrogen bonds, forming infinite chains running along [010]. The crystal structure also features C—H⋯O and π–π stacking inter­actions, which assemble the chains into supra­molecular layers parallel to (100). The π–π stacking inter­actions are observed between the pyridine rings of inversion-related cations with a centroid–centroid distance of 3.867 (2) Å.

## Related literature

For co-crystallization of pharmaceuticals, see: Vishweshwar *et al.* (2006 ▶); Lemmerer (2012 ▶). For the crystal structures of related compounds, see: Ośmiałowski *et al.* (2010*b* ▶); Aakeröy *et al.* (2006 ▶, 2010 ▶). For the role of steric effects in hydrogen-bonded compounds, see Ośmiałowski *et al.* (2012*a* ▶,*b* ▶, 2010*a* ▶,*b* ▶). For the synthesis of 2-pivaloyl­amino-6-amino­pyridine, see: Ośmiałowski *et al.* (2010*a* ▶). 
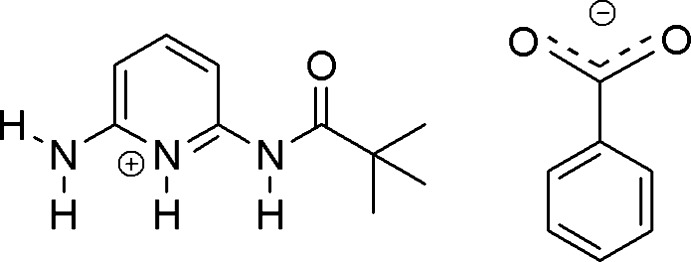


## Experimental

### 

#### Crystal data

C_10_H_16_N_3_O^+^·C_7_H_5_O_2_^−^
*M* *_r_* = 315.37
Monoclinic, 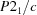

*a* = 15.1438 (4) Å
*b* = 5.7099 (2) Å
*c* = 18.7388 (6) Å
β = 91.967 (2)°
*V* = 1619.38 (9) Å^3^
*Z* = 4
Mo *K*α radiation
μ = 0.09 mm^−1^
*T* = 123 K
0.13 × 0.10 × 0.08 mm

#### Data collection

Bruker–Nonius KappaCCD diffractometer with APEXII detector
Absorption correction: multi-scan (*SADABS*; Sheldrick, 2004 ▶) *T*_min_ = 0.988, *T*_max_ = 0.993
10993 measured reflections
3724 independent reflections
2054 reflections with *I* > 2σ(*I*)
*R*_int_ = 0.094

#### Refinement

*R*[*F* ^2^ > 2σ(*F* ^2^)] = 0.066
*wR*(*F* ^2^) = 0.141
*S* = 1.00
3724 reflections
220 parameters
4 restraints
H-atom parameters constrained
Δρ_max_ = 0.24 e Å^−3^
Δρ_min_ = −0.28 e Å^−3^

### 

Data collection: *COLLECT* (Bruker, 2008 ▶); cell refinement: *DENZO-SMN* (Otwinowski & Minor, 1997 ▶); data reduction: *DENZO-SMN*; program(s) used to solve structure: *SIR2004* (Burla *et al.*, 2005 ▶); program(s) used to refine structure: *SHELXL2013* (Sheldrick, 2008 ▶); molecular graphics: *PLATON* (Spek, 2009 ▶); software used to prepare material for publication: *SHELXL2013* and *publCIF* (Westrip, 2010 ▶).

## Supplementary Material

Crystal structure: contains datablock(s) I, global. DOI: 10.1107/S1600536813023787/fy2103sup1.cif

Structure factors: contains datablock(s) I. DOI: 10.1107/S1600536813023787/fy2103Isup2.hkl

Supplementary material file. DOI: 10.1107/S1600536813023787/fy2103Isup3.cml

Additional supplementary materials:  crystallographic information; 3D view; checkCIF report

## Figures and Tables

**Table 1:** Hydrogen-bond geometry (Å, °)

*D*—H⋯*A*	*D*—H	H⋯*A*	*D*⋯*A*	*D*—H⋯*A*
N1—H1⋯O13*A* ^i^	0.91 (2)	1.67 (2)	2.571 (2)	170 (2)
N6—H6*A*⋯O13*B* ^i^	0.90 (2)	2.05 (2)	2.934 (3)	167 (2)
N6—H6*B*⋯O13*B* ^ii^	0.91 (2)	2.05 (2)	2.869 (3)	149 (2)
N7—H7⋯O13*A* ^i^	0.86 (2)	2.24 (2)	2.984 (3)	146 (2)
C4—H4⋯O8^iii^	0.95	2.49	3.433 (3)	172

Symmetry codes: (i) 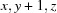; (ii) 
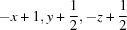
; (iii) 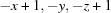.

**Fig. 1.**
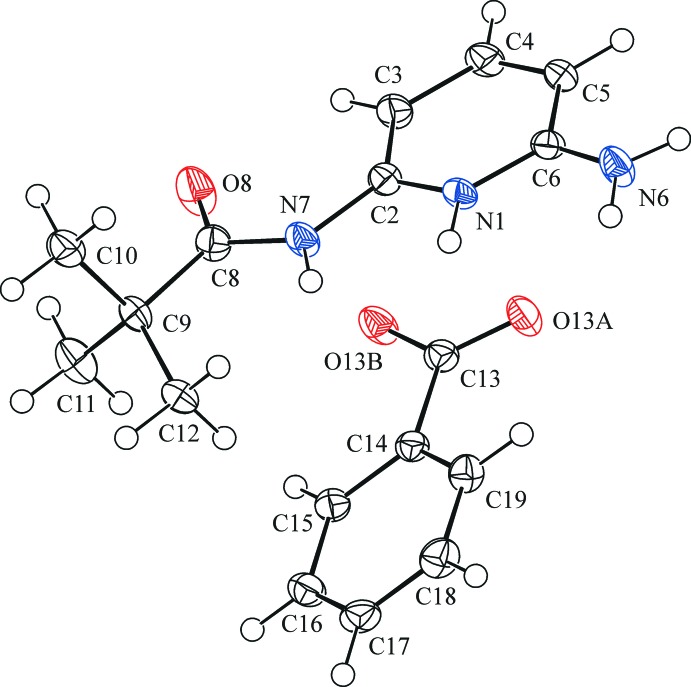
The molecular structure of (I), with atom labels and 50% probability displacement ellipsoids for non-H atoms.

**Fig. 2.**
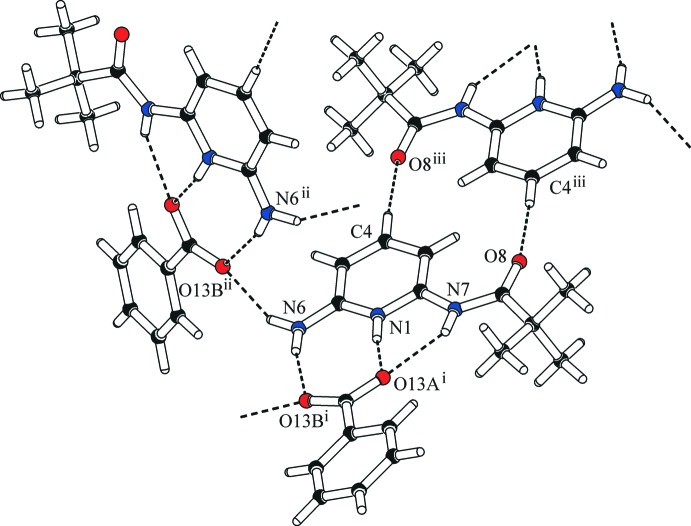
A part of the crystal structure of (I), showing the intermolecular interactions as dashed lines [symmetry codes: (i) x,y + 1,z; (ii)-x + 1,y + 1/2,-z + 1/2; (iii)-x + 1,- y,-z + 1].

## supplementary crystallographic information

### 1. Comment

Co-crystallization is used in the pharmaceutical industry to improve the shelf life of drugs (Vishweshwar *et al.*, 2006; Lemmerer, 2012). It is also used in many fields of chemistry, including material science. It is known that 2-acylaminopyridine forms co-crystals with acids, while in 2-aminopyridine acid complexes proton transfer takes place, yielding salts (Aakeröy *et al.*, 2010; 2006). The current report deals with the competition between formation of a salt and co-crystal. It is worth pointing out that the 2-acylamino moiety prefers to form co-crystals, while the 6-amino moiety prefers salt formation. In 2-pivaloylamino pyridine, both groups are present in the same molecule. Moreover, the increased acidity of NH in the –NHCO– group, in general, increases the hydrogen bonding donation ability of the NH proton. On the other hand, we used the sterically demanding pivaloyl group to hinder the efficient NH···O═C interaction of the —NHCO—*t*Bu part of the title molecule. Thus the interacting acid is pushed to transfer the proton to the heterocyclic nitrogen and to form a salt with 2-pivaloylamino-6-aminopyridine. It is worth noting that the NH_2_ group attached to C6 of the pyridine ring causes an increase of electron density at the ring nitrogen. More systematic studies on co-crystallization of 2-acylaminopyridine with benzoic acids are in progress. For the steric effects in hydrogen bonded compounds, refer to our previous publications (Ośmiałowski *et al.*, 2012*a*,*b*; 2010*a*,*b*).

As illustrated in Figure 1, the asymmetric unit of the title salt, (I), contains one protonated 2-pivaloylamino-6-aminopyridine cation and one benzoate anion, both located in general positions.

The geometric parameters of the 2-pivaloylamino-6-aminopyridine cation are in good agreement with those found for the related structures (Ośmiałowski *et al.*, 2010*a*,*b*). In the benzoate anion the C—O distances, 1.268 (3) Å and 1.253 (3) Å, clearly indicate the delocalization of the negative charge within the carboxylate group.

In the crystal of the title salt, cations and anions are connected *via* four N—H···O hydrogen bonds (Table 1 and Figure 2). The protonated N1 atom and two nitrogen atoms (N6 and N7) interact with the carboxylate oxygen atoms (O13A and O13B; symmetry code (i): *x*, *y* + 1, *z*) and form hydrogen-bonded aggregates. Such structural motifs are further propagated into infinite chains running along *b* axis by N6—H6B···O13B^ii^ [symmetry code (ii): -*x* + 1, *y* + 1/2, -*z* + 1/2] hydrogen bond. The crystal structure of (I) is further stabilized by an almost linear C4—H4···O8^iii^ interaction (Table 1 and Figure 2) and by π···π stacking interactions; both of which connect the adjacent one-dimensional-chains to produce (100) supramolecular sheets. The thickness of each separate layer is equal to the *a* unit cell constant. No direction-specific interactions have been found between the supramolecular sheets.

The aforementioned π···π stacking interactions are observed between the pyridine rings of inversion-related cations, with a *Cg*···*Cg*^iv^ distance of 3.867 (2) Å and interplanar distance of 3.455 (1) Å; *Cg* is the centroid of the N2/C2–C6 ring, symmetry code (iv): 1 - *x*, 1 - *y*, 1 - *z*.

### 2. Experimental

For the synthesis of the title compound, equimolar ammounts of 2-pivaloylamino-6-aminopyridine and benzoic acid were mixed in methanol. The solution was left for a couple of days for slow evaporation and produced single crystals. The parent 2-pivaloylamino-6-aminopyridine was prepared according to a literature procedure (Ośmiałowski *et al.*, 2010*a*).

### 3. Refinement

All non-hydrogen atoms were refined anisotropically. H atoms bonded to N atoms were located in a difference map and refined with distance restraints of N1—H1 (and N7—H7) = 0.88 (2) Å, N6—H6A (and N6—H6B) = 0.91 (2) Å and with *U*_iso_(H) = 1.2*U*_eq_(N). Other H atoms were positioned geometrically and refined using a riding model with C—H = 0.95–0.98 Å and with *U*_iso_(H) = 1.2 *U*_eq_(C) or 1.5 *U*_eq_(methyl C).

### Crystal data

**Table:**

C_10_H_16_N_3_O^+^·C_7_H_5_O_2_^−^	*F*(000) = 672
*M**_r_* = 315.37	*D*_x_ = 1.293 Mg m^−^^3^
Monoclinic, *P*2_1_/*c*	Mo *K*α radiation, λ = 0.71073 Å
*a* = 15.1438 (4) Å	Cell parameters from 4257 reflections
*b* = 5.7099 (2) Å	θ = 0.4–28.3°
*c* = 18.7388 (6) Å	µ = 0.09 mm^−^^1^
β = 91.967 (2)°	*T* = 123 K
*V* = 1619.38 (9) Å^3^	Block, colourless
*Z* = 4	0.13 × 0.10 × 0.08 mm

### Data collection

**Table:**

Bruker–Nonius KappaCCD diffractometer with APEXII detector	3724 independent reflections
Radiation source: fine-focus sealed tube	2054 reflections with *I* > 2σ(*I*)
Graphite monochromator	*R*_int_ = 0.094
Detector resolution: 9 pixels mm^-1^	θ_max_ = 27.5°, θ_min_ = 2.5°
φ and ω scans	*h* = −19→18
Absorption correction: multi-scan (*SADABS*; Sheldrick, 2004)	*k* = −7→7
*T*_min_ = 0.988, *T*_max_ = 0.993	*l* = −24→20
10993 measured reflections	

### Refinement

**Table:**

Refinement on *F*^2^	Primary atom site location: structure-invariant direct methods
Least-squares matrix: full	Secondary atom site location: difference Fourier map
*R*[*F*^2^ > 2σ(*F*^2^)] = 0.066	Hydrogen site location: inferred from neighbouring sites
*wR*(*F*^2^) = 0.141	H-atom parameters constrained
*S* = 1.00	*w* = 1/[σ^2^(*F*_o_^2^) + (0.0496*P*)^2^] where *P* = (*F*_o_^2^ + 2*F*_c_^2^)/3
3724 reflections	(Δ/σ)_max_ < 0.001
220 parameters	Δρ_max_ = 0.24 e Å^−^^3^
4 restraints	Δρ_min_ = −0.28 e Å^−^^3^

### Special details

**Table:**

Geometry. All e.s.d.'s (except the e.s.d. in the dihedral angle between two l.s. planes) are estimated using the full covariance matrix. The cell e.s.d.'s are taken into account individually in the estimation of e.s.d.'s in distances, angles and torsion angles; correlations between e.s.d.'s in cell parameters are only used when they are defined by crystal symmetry. An approximate (isotropic) treatment of cell e.s.d.'s is used for estimating e.s.d.'s involving l.s. planes.

### Fractional atomic coordinates and isotropic or equivalent isotropic displacement parameters (Å^2^)

**Table:**

	*x*	*y*	*z*	*U*_iso_*/*U*_eq_	
O8	0.69413 (12)	0.1535 (3)	0.54656 (10)	0.0370 (5)	
N1	0.57982 (12)	0.6015 (4)	0.39112 (11)	0.0205 (5)	
H1	0.6221 (14)	0.710 (4)	0.3842 (13)	0.025*	
N6	0.48943 (14)	0.7758 (4)	0.30603 (12)	0.0278 (5)	
H6A	0.5311 (15)	0.882 (4)	0.2960 (14)	0.033*	
H6B	0.4404 (14)	0.774 (4)	0.2764 (13)	0.033*	
N7	0.68521 (13)	0.4637 (4)	0.47095 (11)	0.0231 (5)	
H7	0.7141 (15)	0.580 (4)	0.4546 (13)	0.028*	
C2	0.60154 (15)	0.4307 (4)	0.43895 (13)	0.0223 (6)	
C3	0.54405 (15)	0.2505 (4)	0.45137 (14)	0.0249 (6)	
H3	0.5582	0.1307	0.4850	0.030*	
C4	0.46378 (16)	0.2513 (4)	0.41222 (14)	0.0261 (6)	
H4	0.4225	0.1295	0.4200	0.031*	
C5	0.44278 (15)	0.4204 (4)	0.36334 (13)	0.0227 (6)	
H5	0.3881	0.4149	0.3369	0.027*	
C6	0.50276 (15)	0.6023 (4)	0.35252 (13)	0.0209 (6)	
C8	0.72715 (16)	0.3289 (4)	0.52242 (14)	0.0234 (6)	
C9	0.81731 (15)	0.4228 (4)	0.54969 (13)	0.0219 (6)	
C10	0.79992 (16)	0.5515 (5)	0.61949 (14)	0.0293 (6)	
H10A	0.7718	0.4443	0.6526	0.044*	
H10B	0.7608	0.6852	0.6096	0.044*	
H10C	0.8560	0.6072	0.6409	0.044*	
C11	0.87823 (17)	0.2125 (4)	0.56515 (16)	0.0326 (7)	
H11A	0.8896	0.1308	0.5204	0.049*	
H11B	0.8497	0.1049	0.5980	0.049*	
H11C	0.9342	0.2675	0.5869	0.049*	
C12	0.86140 (15)	0.5877 (5)	0.49737 (14)	0.0263 (6)	
H12A	0.8723	0.5037	0.4529	0.040*	
H12B	0.9176	0.6436	0.5185	0.040*	
H12C	0.8225	0.7216	0.4871	0.040*	
O13A	0.71169 (10)	−0.1201 (3)	0.37777 (10)	0.0287 (5)	
O13B	0.64016 (11)	0.0950 (3)	0.29481 (10)	0.0289 (5)	
C13	0.70775 (15)	0.0432 (4)	0.33191 (13)	0.0215 (6)	
C14	0.79105 (15)	0.1829 (4)	0.32285 (13)	0.0203 (6)	
C15	0.87128 (15)	0.0994 (4)	0.35068 (13)	0.0220 (6)	
H15	0.8731	−0.0436	0.3766	0.026*	
C16	0.94848 (16)	0.2232 (4)	0.34092 (14)	0.0255 (6)	
H16	1.0031	0.1649	0.3600	0.031*	
C17	0.94615 (16)	0.4311 (4)	0.30351 (14)	0.0271 (6)	
H17	0.9992	0.5155	0.2966	0.033*	
C18	0.86638 (16)	0.5174 (5)	0.27596 (14)	0.0279 (6)	
H18	0.8647	0.6621	0.2509	0.033*	
C19	0.78894 (16)	0.3920 (4)	0.28499 (13)	0.0245 (6)	
H19	0.7345	0.4494	0.2652	0.029*	

### Atomic displacement parameters (Å^2^)

**Table:**

	*U*^11^	*U*^22^	*U*^33^	*U*^12^	*U*^13^	*U*^23^
O8	0.0319 (11)	0.0331 (11)	0.0450 (13)	−0.0155 (8)	−0.0117 (9)	0.0167 (10)
N1	0.0153 (11)	0.0213 (11)	0.0248 (12)	−0.0069 (8)	0.0000 (8)	0.0002 (9)
N6	0.0206 (12)	0.0284 (13)	0.0339 (14)	−0.0070 (9)	−0.0076 (10)	0.0059 (11)
N7	0.0175 (11)	0.0242 (12)	0.0275 (13)	−0.0068 (9)	−0.0028 (9)	0.0046 (10)
C2	0.0197 (13)	0.0264 (14)	0.0208 (14)	−0.0036 (10)	0.0006 (10)	−0.0012 (11)
C3	0.0208 (13)	0.0273 (15)	0.0266 (15)	−0.0069 (10)	0.0002 (11)	0.0067 (11)
C4	0.0202 (14)	0.0284 (14)	0.0297 (16)	−0.0106 (11)	0.0016 (11)	−0.0020 (12)
C5	0.0166 (12)	0.0281 (14)	0.0234 (14)	−0.0049 (10)	−0.0005 (10)	−0.0004 (11)
C6	0.0165 (12)	0.0254 (14)	0.0210 (14)	−0.0004 (10)	0.0029 (10)	−0.0029 (11)
C8	0.0235 (13)	0.0221 (14)	0.0246 (15)	−0.0043 (11)	−0.0006 (11)	0.0012 (11)
C9	0.0172 (12)	0.0227 (13)	0.0255 (14)	−0.0018 (10)	−0.0039 (10)	−0.0019 (11)
C10	0.0215 (13)	0.0354 (16)	0.0309 (15)	−0.0046 (11)	−0.0012 (11)	−0.0032 (13)
C11	0.0264 (15)	0.0257 (16)	0.0449 (19)	0.0005 (11)	−0.0076 (12)	0.0017 (13)
C12	0.0171 (13)	0.0323 (15)	0.0295 (15)	−0.0046 (11)	−0.0007 (10)	−0.0001 (12)
O13A	0.0198 (9)	0.0276 (10)	0.0384 (12)	−0.0058 (7)	−0.0032 (8)	0.0102 (9)
O13B	0.0199 (9)	0.0326 (11)	0.0335 (11)	−0.0030 (7)	−0.0069 (8)	0.0078 (9)
C13	0.0201 (13)	0.0225 (14)	0.0220 (14)	−0.0029 (10)	0.0016 (10)	−0.0010 (12)
C14	0.0186 (13)	0.0227 (14)	0.0197 (14)	−0.0027 (10)	0.0022 (10)	−0.0021 (11)
C15	0.0214 (13)	0.0219 (13)	0.0227 (14)	−0.0017 (10)	0.0019 (10)	0.0010 (11)
C16	0.0192 (13)	0.0288 (15)	0.0287 (15)	−0.0016 (10)	0.0012 (10)	−0.0017 (12)
C17	0.0251 (14)	0.0302 (15)	0.0264 (15)	−0.0118 (11)	0.0062 (11)	−0.0010 (12)
C18	0.0329 (15)	0.0231 (14)	0.0278 (15)	−0.0058 (11)	0.0025 (12)	0.0051 (12)
C19	0.0264 (14)	0.0230 (14)	0.0242 (14)	−0.0012 (11)	−0.0008 (11)	0.0010 (12)

### Geometric parameters (Å, º)

**Table:**

O8—C8	1.214 (3)	C10—H10B	0.9800
N1—C6	1.352 (3)	C10—H10C	0.9800
N1—C2	1.358 (3)	C11—H11A	0.9800
N1—H1	0.905 (16)	C11—H11B	0.9800
N6—C6	1.330 (3)	C11—H11C	0.9800
N6—H6A	0.899 (17)	C12—H12A	0.9800
N6—H6B	0.913 (16)	C12—H12B	0.9800
N7—C8	1.373 (3)	C12—H12C	0.9800
N7—C2	1.396 (3)	O13A—C13	1.268 (3)
N7—H7	0.859 (17)	O13B—C13	1.253 (3)
C2—C3	1.373 (3)	C13—C14	1.507 (3)
C3—C4	1.398 (3)	C14—C19	1.389 (3)
C3—H3	0.9500	C14—C15	1.390 (3)
C4—C5	1.361 (3)	C15—C16	1.384 (3)
C4—H4	0.9500	C15—H15	0.9500
C5—C6	1.400 (3)	C16—C17	1.379 (4)
C5—H5	0.9500	C16—H16	0.9500
C8—C9	1.538 (3)	C17—C18	1.388 (4)
C9—C12	1.529 (3)	C17—H17	0.9500
C9—C10	1.531 (3)	C18—C19	1.389 (3)
C9—C11	1.536 (3)	C18—H18	0.9500
C10—H10A	0.9800	C19—H19	0.9500
			
C6—N1—C2	122.7 (2)	C9—C10—H10C	109.5
C6—N1—H1	121.5 (16)	H10A—C10—H10C	109.5
C2—N1—H1	115.6 (16)	H10B—C10—H10C	109.5
C6—N6—H6A	123.2 (17)	C9—C11—H11A	109.5
C6—N6—H6B	119.4 (17)	C9—C11—H11B	109.5
H6A—N6—H6B	116 (2)	H11A—C11—H11B	109.5
C8—N7—C2	128.1 (2)	C9—C11—H11C	109.5
C8—N7—H7	117.2 (17)	H11A—C11—H11C	109.5
C2—N7—H7	114.7 (17)	H11B—C11—H11C	109.5
N1—C2—C3	120.7 (2)	C9—C12—H12A	109.5
N1—C2—N7	112.5 (2)	C9—C12—H12B	109.5
C3—C2—N7	126.9 (2)	H12A—C12—H12B	109.5
C2—C3—C4	117.0 (2)	C9—C12—H12C	109.5
C2—C3—H3	121.5	H12A—C12—H12C	109.5
C4—C3—H3	121.5	H12B—C12—H12C	109.5
C5—C4—C3	122.3 (2)	O13B—C13—O13A	124.6 (2)
C5—C4—H4	118.9	O13B—C13—C14	118.9 (2)
C3—C4—H4	118.9	O13A—C13—C14	116.5 (2)
C4—C5—C6	119.1 (2)	C19—C14—C15	119.4 (2)
C4—C5—H5	120.4	C19—C14—C13	120.5 (2)
C6—C5—H5	120.4	C15—C14—C13	120.0 (2)
N6—C6—N1	117.5 (2)	C16—C15—C14	120.4 (2)
N6—C6—C5	124.3 (2)	C16—C15—H15	119.8
N1—C6—C5	118.2 (2)	C14—C15—H15	119.8
O8—C8—N7	122.5 (2)	C17—C16—C15	120.0 (2)
O8—C8—C9	122.4 (2)	C17—C16—H16	120.0
N7—C8—C9	115.0 (2)	C15—C16—H16	120.0
C12—C9—C10	110.1 (2)	C16—C17—C18	120.1 (2)
C12—C9—C11	109.3 (2)	C16—C17—H17	119.9
C10—C9—C11	109.5 (2)	C18—C17—H17	119.9
C12—C9—C8	113.8 (2)	C17—C18—C19	120.0 (2)
C10—C9—C8	105.9 (2)	C17—C18—H18	120.0
C11—C9—C8	108.1 (2)	C19—C18—H18	120.0
C9—C10—H10A	109.5	C14—C19—C18	120.0 (2)
C9—C10—H10B	109.5	C14—C19—H19	120.0
H10A—C10—H10B	109.5	C18—C19—H19	120.0
			
C6—N1—C2—C3	−1.4 (4)	O8—C8—C9—C10	−79.0 (3)
C6—N1—C2—N7	178.4 (2)	N7—C8—C9—C10	98.3 (3)
C8—N7—C2—N1	178.5 (2)	O8—C8—C9—C11	38.3 (3)
C8—N7—C2—C3	−1.8 (4)	N7—C8—C9—C11	−144.3 (2)
N1—C2—C3—C4	0.7 (4)	O13B—C13—C14—C19	12.4 (4)
N7—C2—C3—C4	−179.1 (2)	O13A—C13—C14—C19	−167.2 (2)
C2—C3—C4—C5	0.6 (4)	O13B—C13—C14—C15	−165.7 (2)
C3—C4—C5—C6	−1.1 (4)	O13A—C13—C14—C15	14.7 (3)
C2—N1—C6—N6	−178.5 (2)	C19—C14—C15—C16	0.1 (4)
C2—N1—C6—C5	0.8 (4)	C13—C14—C15—C16	178.2 (2)
C4—C5—C6—N6	179.6 (3)	C14—C15—C16—C17	0.1 (4)
C4—C5—C6—N1	0.4 (4)	C15—C16—C17—C18	0.4 (4)
C2—N7—C8—O8	1.0 (4)	C16—C17—C18—C19	−1.1 (4)
C2—N7—C8—C9	−176.4 (2)	C15—C14—C19—C18	−0.8 (4)
O8—C8—C9—C12	159.9 (2)	C13—C14—C19—C18	−178.9 (2)
N7—C8—C9—C12	−22.8 (3)	C17—C18—C19—C14	1.3 (4)

### Hydrogen-bond geometry (Å, º)

**Table:**

*D*—H···*A*	*D*—H	H···*A*	*D*···*A*	*D*—H···*A*
N1—H1···O13*A*^i^	0.91 (2)	1.67 (2)	2.571 (2)	170 (2)
N6—H6*A*···O13*B*^i^	0.90 (2)	2.05 (2)	2.934 (3)	167 (2)
N6—H6*B*···O13*B*^ii^	0.91 (2)	2.05 (2)	2.869 (3)	149 (2)
N7—H7···O13*A*^i^	0.86 (2)	2.24 (2)	2.984 (3)	146 (2)
C4—H4···O8^iii^	0.95	2.49	3.433 (3)	172

Symmetry codes: (i) *x*, *y*+1, *z*; (ii) −*x*+1, *y*+1/2, −*z*+1/2; (iii) −*x*+1, −*y*, −*z*+1.
